# TMEM138: From Biological Functions to Diseases

**DOI:** 10.33549/physiolres.935479

**Published:** 2025-04-01

**Authors:** Qinghan SHI, Lingling ZHU, Lan ZHANG, Zhaolun GUO, Yafei HAO, Yongmei WANG, Jiangang GAO, Hua LI, Min LIU

**Affiliations:** 1 Department of Gynecology, The Affiliated Taian City Central Hospital of Qingdao University, Taian, China; 2 School of Basic Medical Sciences, Shandong University, Jinan, China; 3 Department of Pain, The Affiliated Taian City Central Hospital of Qingdao University, Taian, China; 4 Medical Science and Technology Innovation Center, Shandong First Medical University, Jinan, China

**Keywords:** TMEM138, Transmembrane protein, Cellular signaling, Neurodegenerative diseases, Tumors

## Abstract

Transmembrane Protein 138 (TMEM138) is a transmembrane protein belonging to the superfamily of transmembrane proteins. Recent research has unveiled its involvement in various biological processes such as cell proliferation, differentiation, and apoptosis. Furthermore, abnormal expression of TMEM138 has been linked to a range of diseases, particularly neurodegenerative diseases and tumors. This review provides an overview of TMEM138, focusing on its molecular characteristics, biological functions, and potential roles in disease pathogenesis. The aim is to offer a valuable reference for future research and clinical applications.

## Introduction

TMEM138, a transmembrane protein, was initially linked to Joubert syndrome (JS) [1]. Further investigation into TMEM138 has sparked interest in its involvement in cellular physiological processes. Research indicates that TMEM138 may influence cell proliferation, differentiation, and apoptosis through intracellular signal transduction pathways. Moreover, dysregulated TMEM138 expression is implicated in various diseases, particularly neurodegenerative disorders and certain tumors. This article succinctly reviews recent advancements in understanding the molecular characteristics, biological functions, and research methodologies related to TMEM138, aiming to inspire further exploration of its role in relevant diseases.

## Molecular characteristics of TMEM138

The TMEM138 gene, located at the 11q12.2 locus on human chromosomes, consists of 7 exons. Protein encoding begins from exon 2, resulting in a trimeric membrane protein with a molecular weight of 18.4kDa and 162 amino acids [2,3]. Across different species, TMEM138 exhibits conserved structures with highly conserved amino acids, motifs, and spatial conformations at primary, secondary, and tertiary levels. Its protein function is relatively conserved, aligning with biological evolution [4]. The transmembrane domain of TMEM138 plays a crucial role in functional conservation.

**Fig. 1:**
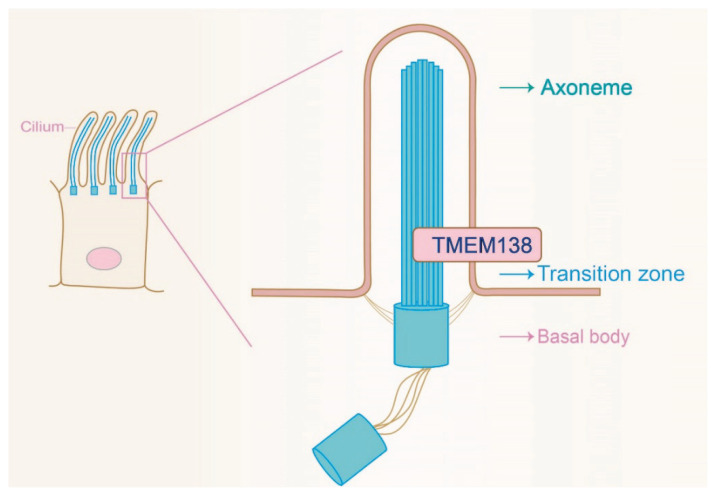
The localization of TMEM138 in cilia. Tmem138 is localized in the ciliary transition zone (TZ).

Experimental evidence has confirmed the cellular localization of TMEM138 in the ciliary transition zone (TZ) (Fig. 1) [5,6]. Studies have shown that TMEM138 is present in the ciliary axoneme and base of cilia in inner medullary collecting duct epithelial cells (IMCD3) of mouse kidneys and in the sensory neuron TZ of Caenorhabditis elegans [3]. Guo *et al*. discovered TMEM138’s localization near the proximal end of the connecting cilium (CC) in retinal photoreceptors, closely associated with the distal ciliary cytoplasmic protein Ahi1. This relationship suggests that TMEM138 may play a role in transporting outer segment proteins alongside Ahi1 [2].

Expression Pattern TMEM138 is detected in various tissues, such as the testes and the nervous system. This broad expression pattern highlights the significant role of this gene in diverse biological processes. Adjacent genes in the genome often exhibit similar expression patterns (co-expression) within cis-regulatory modules. Previous studies have shown that TMEM138 and TMEM216 are positioned head-to-tail in the JBTS2 region of chromosome 11, with their cooperative function mediated by the RFX4 protein. Mutations in TMEM138 can result in a JS phenotype that is indistinguishable from that caused by TMEM216 mutations, despite the lack of sequence homology between the two genes. Notably, their expression is controlled by a conserved regulatory sequence in the non-coding region between them [3,7]. Lee *et al*. utilized microarray databases and in situ hybridization to identify the tissue expression patterns of human TMEM138 and TMEM216, revealing co-expression in major tissues like the brain and kidneys, as well as similar expression profiles in various tissues of human embryos aged 4 to 8 weeks [3]. Through qPCR analysis of coordinated expression levels in mice and zebrafish, it was observed that TMEM138 and TMEM216 share regulatory elements within a gene interval of approximately 23Kb. Importantly, mutations in either of these genes can lead to indistinguishable ciliopathy phenotypes [8,9].

## Biological functions of TMEM138

The TMEM138 gene encodes a protein with multiple transmembrane domains that is expressed in various tissues and involved in regulating biological processes. While its exact cellular localization and function are not fully understood, studies suggest it may impact neural development and disease occurrence by influencing intracellular signal transduction pathways, cytoskeleton dynamics, and membrane protein transport mechanisms. TMEM138 has been identified as a potential tumor suppressor gene for lung cancer, with links to Notch, Hippo, MAPK, and other signaling pathways related to cell proliferation, differentiation, and apoptosis [10].

Research also indicates that RFX2 is important for spermatogenesis and ciliary maturation, with connections to various functions like cell cycle, sperm development, and ciliary assembly. And correlation analysis of protein and transcriptome data shows that RFX2 is significantly correlated with FOXJ1, DNAH9, TMEM138, and so forth [11–13]. As a key protein for ciliary transport and biogenesis, TMEM138 plays a crucial role in signal transduction processes due to its unique characteristics [14]. Mutations in TMEM138 may lead to abnormalities in axons and signal transduction, emphasizing the need for further research to understand its molecular functions and mechanisms in various diseases.

## TMEM138 and disease

**Fig. 2:**
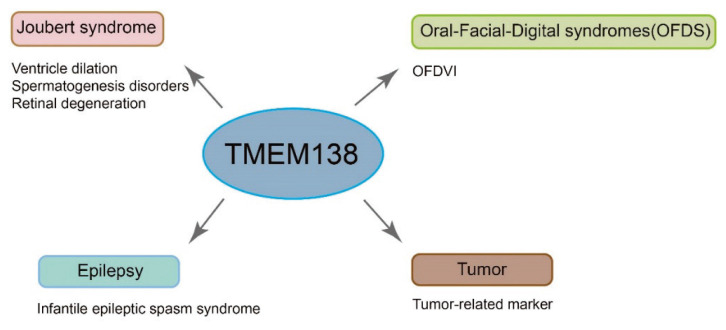
TMEM138 and Disease. TMEM138 gene deletion can lead to a ciliopathy called Joubert syndrome, which affects the brain, retina, and reproductive system. Furthermore, TMEM138 has been linked to a variety of disorders including nervous system malformations, epilepsy, oral-facial-digital syndrome, and tumors.

Transmembrane proteins, acting as channel proteins on biological membranes, play a crucial physiological role. Research has shown that transmembrane proteins are linked to Parkinson’s disease, other neurodegenerative diseases, and malignant tumors [15–18]. Among the transmembrane protein family, TMEM175 deficiency has been associated with an increased risk of Parkinson’s disease [19]. In the context of malignant tumors, transmembrane proteins can modulate tumor occurrence and progression by influencing cell proliferation, migration, invasion, and epithelial-mesenchymal transition [20,21]. The expression levels of TMEM138 are also correlated with disease severity. Deletion of the TMEM138 gene leads to phenotypes like ventricle dilation, spermatogenesis disorders, and retinal degeneration, which are linked to JS [2]. Studies have revealed that TMEM138 is widely expressed in the nervous system and participates in various neurodevelopmental processes. Deficiency or mutation of TMEM138 is associated with abnormal neuronal development and malformations in brain structure, impacting nervous system function. JS is a common congenital neurodevelopmental disorder related to cilia, characterized by structural or functional abnormalities in primary cilia or dysregulation of cilia length. Clinical features of JS include cerebellar vermis hypoplasia, decreased muscle tone, developmental delay, intellectual disability, abnormal eye movements, and the characteristic ‘molar tooth sign’ (MTS) on neuroimaging, indicating midbrain-hindbrain malformation [22–24]. JS exhibits significant genetic heterogeneity, with over 40 gene mutations currently identified as associated with the syndrome. These genes encode proteins that form a functional complex crucial for the structure and function of primary cilia, with TMEM138 being one of them [25]. Bizzari *et al*. documented a case of JS potentially linked to a TMEM138 mutation, where the patient exhibited global developmental delay at 17 months, along with clinical features like nystagmus, strabismus, undescended testes, and imaging findings indicating renal tubular interstitial nephritis with medullary cystic kidney disease [1]. Furthermore, TMEM138 is implicated in various other conditions such as nervous system malformations, epilepsy, oral-facial-digital syndrome, and tumors. Research has shown that mutations in TMEM138 play a significant role in the pathogenesis of these disorders. For instance, Pan *et al*. identified TMEM138 among 1739 epilepsy-related genes, highlighting its importance in infantile epileptic spasm syndrome. Additionally, mutations in TMEM138 and other genes have been associated with Meckel-Gruber syndrome and type VI oral-facial-digital syndrome [26–28] (Fig. 2).

Recent research increasingly supports the significant role of primary cilia in the regulation of tumor development[29]. Studies have demonstrated that primary cilia play a crucial role in the carcinogenesis process by influencing cell cycle regulation, autophagy, and various signaling pathways [30,31]. Tumors represent a major health concern due to their high incidence and mortality rates, challenging early diagnosis, poor prognosis, and tendency for recurrence. Understanding the mechanisms underlying tumor formation is essential for developing improved treatment strategies and slowing down malignant progression. Identification of tumor-related biomarkers can facilitate early tumor detection and the design of molecular targeted therapies [32–34]. Zhao *et al*. utilized a combination of omics data to develop bioinformatics approaches for identifying biomarkers associated with tumor diagnosis and prognosis. Through this method, they identified 10 prognostic biomarkers for breast invasive cancer, including TMEM138 [35]. Additionally, Zhao and colleagues employed CRISPR/Cas9 knockout library technology to conduct a whole-genome screening, identifying 38 potential tumor suppressor genes for lung cancer, with TMEM138 being one of them. Notably, the expression of the TMEM138 gene was found to be positively correlated with the occurrence of lung squamous cell carcinoma [10]. Further exploration of TMEM138 may lead to its potential as a novel tumor-related marker for predicting patient prognosis and enabling personalized tumor treatment.

## Research methods for TMEM138

Several research methods are commonly used to study the TMEM138 gene. Gene knockout technology, including traditional methods like homologous recombination and emerging methods like CRISPR-Cas9, allows for targeted knockout of the gene[36]. Guo *et al*. utilized a TMEM138 gene knockout mouse model to investigate its role in retinal diseases, revealing disruptions in outer segment morphogenesis and rapid retinal degeneration [2]. Lee *et al*. demonstrated similar effects by knocking out TMEM138 in zebrafish, resulting in ventricle expansion [3]. Protein interaction analysis is another valuable method, as proteins play a crucial role in cellular functions and disease pathogenesis. Understanding protein interactions can provide insights into potential drug targets [37,38]. Various methods have been developed to detect protein interactions, such as the yeast two-hybrid system [39], co-immunoprecipitation [40], pull-down [41], and proximity labeling techniques [42]. Guo *et al*. investigated the role of TMEM138 in photoreceptor outer segments through co-immuno-precipitation and retinal pull-down experiments, revealing direct interactions between TMEM138, Ahi, and Rhodopsin [2]. Additionally, they discovered an interaction between the membrane protein TMEM231, TMEM138, and Rhodopsin, with abnormal ciliary membrane localization of TMEM231 in TMEM138 mutant mice.

## Future research directions

Future research directions include further exploration of the localization and ciliary function of TMEM138 in humans and mice, as well as investigating its role in the reproductive system. Mutations in TMEM138 have been linked to azoospermia and infertility, highlighting the need for a deeper understanding of its mechanisms for potential diagnostic and therapeutic advancements in reproductive system diseases. The TMEM family members have been associated with tumors and can serve as prognostic biomarkers. However, the exact role of TMEM138 in tumor regulation remains unclear, necessitating further exploration of gene mutations and related signaling pathways. Future research should focus on investigating the impact of TMEM138 on tumor occurrence and progression, as well as its potential for developing novel treatment methods and identifying new targets for tumor prevention, diagnosis, and treatment.

## Conclusion

In conclusion, TMEM138, a versatile transmembrane protein, plays a crucial role in cellular physiology and pathology, particularly in cilia-related diseases, cell cycle regulation, sperm development, and signal transduction. Mutations in TMEM138 can result in severe neurodevelopmental disorders like Joubert syndrome. Recent scholarly attention to TMEM138 highlights the importance of studying its function and regulatory mechanisms, offering insights into disease pathogenesis and paving the way for innovative diagnostic and therapeutic approaches.
